# CaWRKY22 Acts as a Positive Regulator in Pepper Response to *Ralstonia* *Solanacearum* by Constituting Networks with CaWRKY6, CaWRKY27, CaWRKY40, and CaWRKY58

**DOI:** 10.3390/ijms19051426

**Published:** 2018-05-10

**Authors:** Ansar Hussain, Xia Li, Yahong Weng, Zhiqin Liu, Muhammad Furqan Ashraf, Ali Noman, Sheng Yang, Muhammad Ifnan, Shanshan Qiu, Yingjie Yang, Deyi Guan, Shuilin He

**Affiliations:** 1 Ministry of Education Key Laboratory of Plant Genetic Improvement and Comprehensive Utilization, Fujian Agriculture and Forestry University, Fuzhou 350002, China; ahtraggar@yahoo.com (A.H.); lixiatrista@163.com (X.L.); wwwyh0915@163.com (Y.W.); lzqfujian@126.com (Z.L.); furqanashraf@hotmail.com (M.F.A.); alinoman@gcuf.edu.pk (A.N.); yangsheng2061@163.com (S.Y.); mifnan@yahoo.com (M.I.); B.miracle@hotmail.com (S.Q.); hnyingjieyang@163.com (Y.Y.); gdyfujian@126.com (D.G.); 2 College of Crop Science, Fujian Agriculture and Forestry University, Fuzhou 350002, China; 3 Key Laboratory of Applied Genetics of Universities in Fujian Province, Fujian Agriculture and Forestry University, Fuzhou 350002, China; 4 Department of Botany, Government College University, Faisalabad 38040, Pakistan

**Keywords:** *Capsicum annuum*, CaWRKY22, immunity, *Ralstonia Solanacearum*, WRKY networks

## Abstract

The WRKY web, which is comprised of a subset of WRKY transcription factors (TFs), plays a crucial role in the regulation of plant immunity, however, the mode of organization and operation of this network remains obscure, especially in non-model plants such as pepper (*Capsicum annuum*). Herein, *CaWRKY22*, a member of a subgroup of IIe WRKY proteins from pepper, was functionally characterized in pepper immunity against *Ralstonia Solanacearum*. CaWRKY22 was found to target the nuclei, and its transcript level was significantly upregulated by *Ralstonia Solanacearum* inoculation (RSI) and exogenously applied salicylic acid (SA), Methyl jasmonate (MeJA), or ethephon (ETH). Loss-of-function *CaWRKY22*, caused by virus-induced gene silencing (VIGS), enhanced pepper’s susceptibility to RSI. In addition, the silencing of *CaWRKY22* perturbed the hypersensitive response (HR)-like cell death elicited by RSI and downregulated defense-related genes including *CaPO2*, *CaPR4*, *CaACC*, *CaBPR1*, *CaDEF1*, *CaHIR1*, and *CaWRKY40*. CaWRKY22 was found to directly bind to the promoters of *CaPR1*, *CaDEF1*, and *CaWRKY40* by chromatin immuno-precipitation (ChIP) analysis. Contrastingly, transient overexpression of *CaWRKY22* in pepper leaves triggered significant HR-like cell death and upregulated the tested immunity associated maker genes. Moreover, the transient overexpression of *CaWRKY22* upregulated the expression of *CaWRKY6* and *CaWRKY27* while it downregulated of the expression of *CaWRKY58*. Conversely, the transient overexpression of *CaWRKY6*, *CaWRKY27*, and *CaWRKY40* upregulated the expression of *CaWRKY22*, while transient overexpression of *CaWRKY58* downregulated the transcript levels of *CaWRKY22*. These data collectively recommend the role of CaWRKY22 as a positive regulator of pepper immunity against *R. Solanacearum*, which is regulated by signaling synergistically mediated by SA, jasmonic acid (JA), and ethylene (ET), integrating into WRKY networks with WRKY TFs including CaWRKY6, CaWRKY27, CaWRKY40, and CaWRKY58.

## 1. Introduction

Being sessile, plants frequently encounter various biotic and abiotic stresses individually, and in some cases, collectively [1]. To protect against stresses, plants have evolved a sophisticated defense system developed under frequent selection pressures from the main environmental constraints. This system is largely regulated at the transcriptional level by the action of different transcription factors (TFs) interconnected to make a complicated transcriptional networks [2]. Defense responses to different stresses need to be appropriately coordinated and strictly controlled since they are costly to the plants in terms of energy expenditure and development [3]. The defense system might differ in different plant species due to diverse ecological conditions affecting their evolution in the regions/habitats [4]. Therefore, defense mechanisms found in model plants cannot be totally suggested for other non-model plants directly. Plant defense mechanisms have been intensively studied in the past decades, but the majority of these studies have focused on model plants such as *Arabidopsis* and rice. However, the organization of transcriptional networks and their functional coordination to regulate plant responses to different stresses, especially in non-model plants, remains poorly understood.

WRKY proteins constitute one of the largest TF families in plants. WRKY TFs are characterized by one or two conserved WRKY domains and the almost invariant WRKYGQK sequence at the N-terminus followed by a C2H2 or C2HC zinc-finger motif [5]. Based on the number of WRKY domains and the structure of zinc-finger motif, WRKY proteins are phylogenetically classified into three major groups (groups I–III). Group II is further divided into five subgroups (IIa, IIb, IIc, IId, and IIe) [6,7]. By forming a unique wedge shape that inserts perpendicularly into the major groove of the DNA [8], WRKY TFs primarily bind W-boxes [TTGAC(C/T)] present in the promoter regions of target genes through the WRKYGQK motif on the second b-strand, and thereby, transcriptionally modulate the expression of these target genes. By activating or repressing the transcription of their target genes, WRKY TFs have been implicated in plant biological processes like senescence, seed development, dormancy, and germination as well as biotic and abiotic stress responses [9]. It has been found that a subset of WRKY genes was transcriptionally modified by a single stress, or a single WRKY TF participates in multiple stresses. The W-boxes are enriched within the promoter regions [10,11]. These results indicate the existence of WRKY networks involved in plant responses to a specific stress or combined stresses. Although the roles of WRKY proteins in plant responses to biotic and abiotic stresses and their underlying mechanisms have been intensively studied over the years, the majority of these studies have mainly focused on a single gene in the response of plants to a single stress in model plants such as *Arabidopsis* and rice. A significant functional divergence among close structural homologs of WRKY proteins from different plant species was recently suggested [12]. The roles of WRKY TFs and their networks in the response of non-model plants to different single stresses, or to closely related stress combinations, remain poorly understood.

Pepper (*Capsicum annuum*) is a vegetable of great economic importance and is a Solanaceae distributed or planted in uplands during warm seasons, where it is confronted with various soil-borne pathogens, such as *Phytophthora capsici* and *Ralstonia Solanacearum*, the causal agents for pepper blight and bacterial wilt diseases, respectively [13,14]. The coexistence of these soil-borne pathogens causes severe destruction, once the pepper plants experience the combined stress of high temperature and high humidity (HTHH) which attenuates R protein mediated immunity and accelerates the development of pathogens. On the other hand, the combination of pathogen attack and HTHH constitutes most natural selection pressures on pepper that have historically affected its evolution [15]. However, this pressure might not act in the evolution of rice and *Arabidopsis*, which grow in paddy fields during warm seasons or dry lands during cool seasons with fewer soil-borne pathogens. Thus, pepper seems more suitable as a plant for investigation on the coordination of resistance or tolerance against abiotic or biotic stresses, for example, *Ralstonia Solanacearum* inoculation (RSI) and HTHH. Some native pepper varieties from subtropical regions specifically show augmented disease resistance even under HTHH [15]. HSE, a high temperature responsive *cis*-element, was ubiquitously found to co-occur with Salicylic acid (SA)-, Jasmonic acid (JA)-, Ethylene (ET)-, or pathogen-responsive elements in the promoters of the majority of CDPKs and MAPKs, which have been frequently involved in plant immunity [10,11]. This suggests the existence of cross-talk mechanisms between immunity, high temperature and humidity.

Since the genome of pepper is about 27 and 7.5 times larger than that of *Arabidopsis* and rice, respectively, a total of 73 WRKY genes were found in the genome of pepper [16], which is much less than what we expected when compared to the 72 WRKY genes in *Arabidopsis* and 122 in rice [3,6]. Our previous studies indicated that *CaWRKY6* [17], *CaWRKY27* [18], *CaWRKY40* [19], and *CaWRKY58* [20] have been implicated in the pepper response to RSI. Of these, CaWRKY6, CaWRKY27, and CaWRKY40 act as positive regulators, while CaWRKY58 acts as a negative regulator. A subset of W-boxes and HSE elements were found in the promoters of these genes as well as that of other WRKY promoters, implying WRKY networks are involved in pepper’s response to RSI. In addition, CaWRKY40 was also found to be regulated directly by CaWRKY6 [17] and CabZIP63 [21] and indirectly by CaCDPK15 [22]. However, the majority of pepper WRKY TFs have not been characterized in terms of pepper’s response to pathogen infection. In the present study, we report that CaWRKY22, a new IIe WRKY TF of pepper, acts as a positive regulator in pepper’s response to *Ralstonia Solanacearum* inoculation by directly targeting *CaWRKY40* and incorporating a WRKY network including *CaWRKY6*, *CaWRKY27*, *CaWRKY40*, and *CaWRKY58*.

## 2. Results

### 2.1. Cloning and Sequence Analysis of CaWRKY22 cDNA

**Figure 1:**
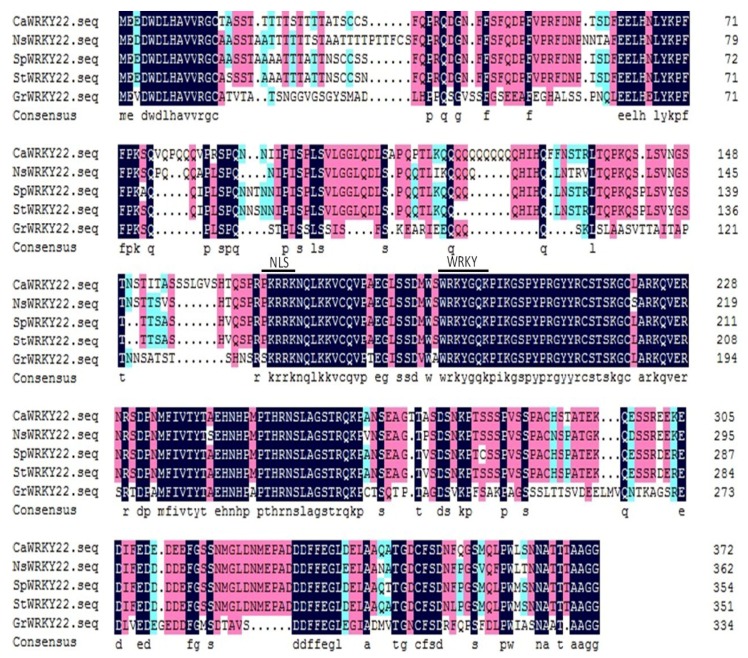
Multiple sequence alignment analysis of proteins related to CaWRKY22. Comparison of the deduced amino acid sequence of CaWRKY22 with that of representative related proteins from *Nicotiana sylvestris* NsWRKY22 (XP009768958.1), *Solanum pennellii* SpWRKY22 (XP015061786.1), *Solanum tuberosum* StWRKY58 (XP006339631.1), and *Gossypium raimondii* GrWRKY22 (XP012490022.1). Green shading, 50–75% identity; red shading, 75–100% identity; black shading, 100% identity. Alignment was carried out by DNAMAN5.

By *cis*-element scanning within the promoters of WRKY genes in the genome sequence of *Capsicum annuum* (http://peppergenome.snu.ac.kr), *CaWRKY22* was selected for further functional characterization. The presence of HSE and immunity associated *cis*-elements such as the TCA, TGACG-motif, and W-box in the *CaWRKY22* promoter region imply its potential role in pepper immunity (Figure S1). By using gene specific primers (Table S1), we cloned a cDNA fragment of *CaWRKY22* (CA08g07730) of 1500 bp in length that contained a 1122 bp open-reading frame (ORF). Its deduced amino acid sequence was 373 amino acid residues in length, containing one conserved WRKY-domain and was classified into subgroup IIe [23] (Figure 1). The size and theoretical pI of the predicted protein were 41.29 kDa and 5.87, respectively. CaWRKY22 shares 91%, 91%, 88%, and 55% of amino acid identities with SpWRKY22, StWRKY22, NsWRKY22, and GrWRKY22, respectively (Figure S2).

### 2.2. The Transcriptional Expression of CaWRKY22 Is Upregulated by R. Solanacearum Infection and Exogenous Applied Phytohormones Including SA, MeJA, and ETH 

**Figure 2:**
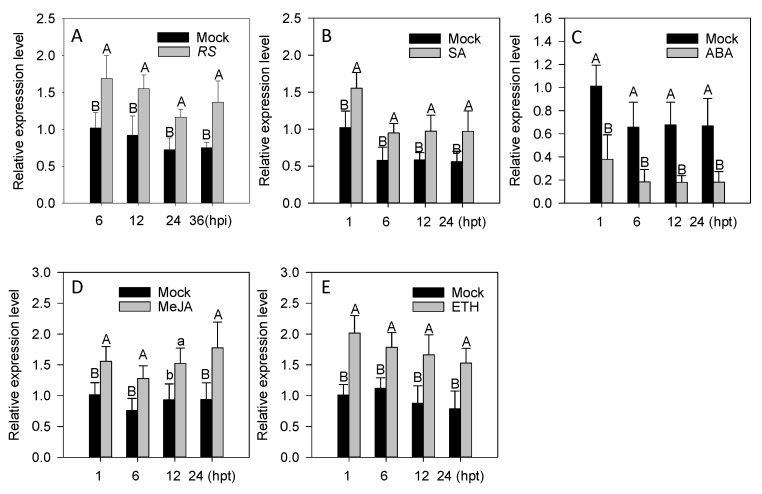
QRT-PCR analysis of relative *CaWRKY22* transcriptional levels in pepper plant leaves exposed to *Ralstonia Solanacearum* inoculation (RSI) and different phytohormones. QRT-PCR was performed to detect the expression levels of *CaWRKY22* in pepper leaves under several treatments including *R. Solanacearum* inoculation, (**A**) application of 1 mM SA; (**B**) application of 100 µm ABA; (**C**) application of 100 µm MeJA; (**D**) and application of 100 µm ETH; (**E**) at different time-points. (**A**) The transcriptional levels in RSI-treated pepper leaves were compared with those in MgCl_2_-treated control plants (mock), whose relative expression level was set to “1”. (**B**–**E**) The transcriptional levels in hormone-treated pepper leaves were compared with those in ddH_2_O-treated plants (mock), whose expression level was set to “1”. Error bars indicated the standard error. Different letters above the bar show a significant difference between the means of the three biological replicates based on the Fisher’s protected LSD test: uppercase letters, *p* < 0.01; lower case letters, *p* < 0.05.

The presence of a subset of putative immunity responsive *cis*-elements in the promoter of *CaWRKY22* implies its inducible expression upon pathogen attack. To test this possibility, qRT-PCR was performed to examine the expression pattern of *CaWRKY22* in response to inoculation of *R. Solanacearum*. The results showed that the transcriptional levels of *CaWRKY22* were upregulated in pepper leaves inoculated with *R. Solanacearum*, compared to that in the mock treated leaves (Figure 2A). The increased *CaWRKY22* transcriptional levels were maintained between 6 and 24 hpi (hours post inoculation) and exhibited maximal levels at 6 hpi, implying the involvement of *CaWRKY22* in the response of pepper toward *R. Solanacearum* (Figure 2A).

Signaling pathways which are mediated by phytohormones such as SA, JA, ET, or ABA (abscisic acid) are involved in the regulation of plant responses to biotic or abiotic stresses. To confirm the data that *CaWRKY22* is involved in the pepper response to RSI, and to test if it is regulated by signaling pathways mediated by these hormones, the relative abundance of *CaWRKY22* against exogenous application of SA, MeJA, ETH, or ABA was measured by qRT-PCR. The results showed that the relative abundance of *CaWRKY22* was enhanced after treatment with 1 mM SA from 1 to 24 hpt (hours post treatment) and exhibited highest levels at 1 hpt (Figure 2B). The exogenous application of ABA resulted in a significant decrease in *CaWRKY22* expression from 1 to 24 hpt (Figure 2C). Similar to the application of SA, treatment of the pepper plant with 100 µM MeJA or 100 µM ETH (ethephon) upregulated the transcription of *CaWRY22* from 1 to 24 hpt compared to the mock treatment (Figure 2D,E).

### 2.3. CaWRKY22 Is Located in the Nuclei

**Figure 3:**
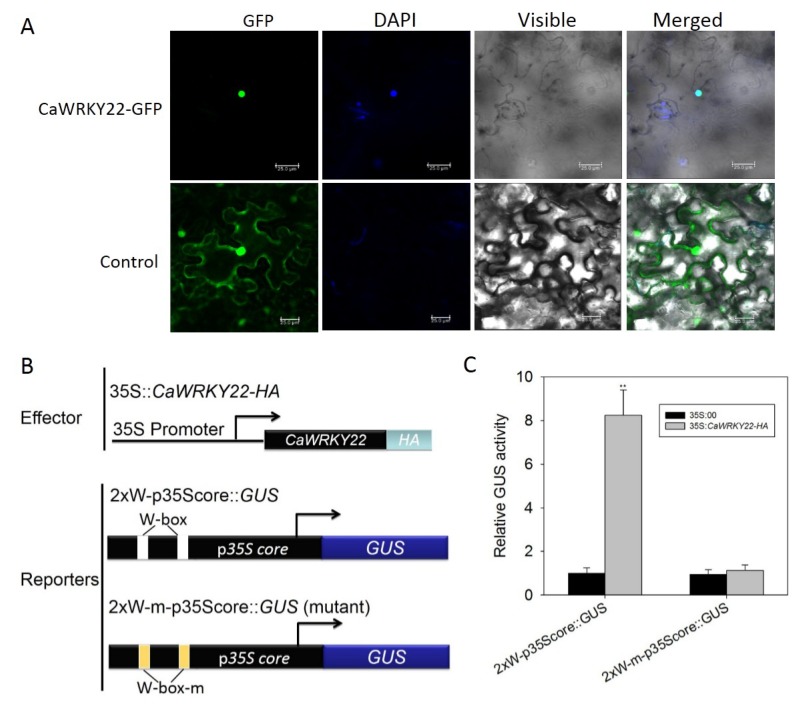
Subcellular localization of CaWRKY22 and its transactivation confirmation experiment. (**A**) CaWRKY22 was exclusively found in the nucleus of *N. benthamiana* leaves, the transiently expressed 35S:*CaWRKY22*–GFP. Green color shows GFP. Blue color shows DAPI staining of nucleus. Cyan clor shows merger of green GFP and DAPI stained nucleus. GFP signal (Green) for the control *N. benthamiana* leaves was found throughout the cell. Images were taken by confocal microscopy at 48 hpi (hours post inoculation). Bars = 25 μm; (**B**) schematic diagram of the effector and reporter constructs used for transient coexpression; (**C**) leaves of pepper were cotransfected with the reporter and effector plasmids and the infiltrated leaves were harvested for GUS activity measurement. The GUS activity of pepper leaves transient coexpressing 2xW-p35Score:GUS and the empty effector vector was set to “1”. Error bars indicated the standard error. The data represents the means ± SD from three biological replicates. The asterisk indicates significant differences, as determined by Fisher’s protected LSD test (* *p* < 0.05, ** *p* < 0.01).

Sequence analysis using WoLFPSORT (“http://www.genscript.com/psort/wolf_psort.html”olf_psort.html) showed that the predicted CaWRKY22 amino acid sequence contains a putative nuclear localization signal (Figure 1), indicating its potential nucleus targeting. To confirm this speculation, we constructed a *CaWRKY22*–GFP fusion construct driven by the constitutive promoter of CaMV35S, and the generated vector was transformed into *Agrobacterium tumefaciens* *strain* GV3101. *CaWRKY22*–GFP was transiently overexpressed in *N. benthamiana* leaves by *Agrobacterium* infiltration, and the GFP signals were observed using a confocal fluorescence microscope. The result showed that the GFP signal of the *CaWRKY22*–GFP was exclusively found in the nuclei, whereas the GFP control was found in multiple subcellular compartments, including the cytoplasm and nuclei (Figure 3A). Several studies have mentioned the binding of WRKY proteins to the W-box manner [TTGAC(C/T)] present in the promoter region of the defense associated target genes, which frequently serve as pathogen responsive regulatory elements. To check whether this also relates to *CaWRKY22*, we conducted a transient coexpression experiment with an effector vector carrying the full length *CaWRKY22* cDNA controlled by a *CaMV35S* promoter (*35S:CaWRK22-HA*), and a reporter vector having *GUS*-control driven by the *CaMV35S* core promoter (−46 to +8 bp), with two copies of W-box (*2xW-p35Score:GUS*) or muted W-box (*2xW-m-p35Score:GUS*) in its proximal upstream region (Figure 3B). Reporter vectors were either transformed individually or co-transformed with the effector construct into *N. benthamiana* leaves by infiltration, and the co-infected leaves were sampled for GUS activity measurement. The GUS quantification result revealed that *N. benthamiana* leaves coinfected with *35S:CaWRK22-HA* and *2xW-p35Score:GUS* exhibited strong GUS activity, compared to the mock leaves, suggesting that CaWRKY22 is capable of active transcription expression of the downstream target gene (Figure 3C).

### 2.4. Effect of CaWRKY22 Loss-of-Function by VIGS on Response of Pepper to R. Solanacearum Inoculation

**Figure 4:**
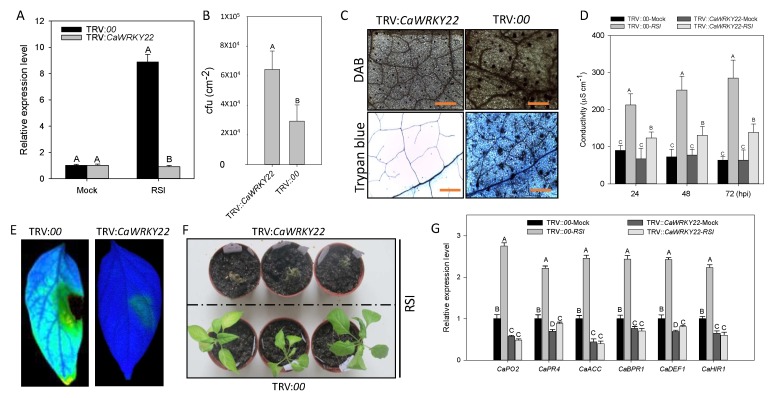
Distinctive responses of *CaWRKY22-*knockout attenuates the pepper’s resistance to RSI. (**A**) QRT-PCR analysis of *CaWRKY22* expression in *R. Solanacearum-*inoculated, mock (inoculated with MgCl_2_ solution) *CaWRKY22-*silenced pepper plants (TRV:*CaWRKY22*), and control plants (TRV:00); (**B**) difference in *R. Solanacearum* growth between *CaWRKY22-*silenced and control pepper plants inoculated with *R. Solanacearum* at 3 dpi (days post inoculation); (**C**) DAB and trypan blue staining in *R. Solanacearum-*inoculated *CaWRKY22-*silenced (TRV:*CaWRKY22*) and control (TRV:00) pepper leaves at 48 hpi. Scale bar = 50 μm; (**D**) electrolyte leakage as ion conductivity to assess the cell death responses in the leaf discs of *CaWRKY22-*silenced (TRV:*CaWRKY22*) and control (TRV:00) pepper after 24, 48, and 72 h of inoculation with and without *R. Solanacearum*; (**E**) cell death in *R. Solanacearum* inoculated *CaWRKY22-*silenced (TRV:*CaWRKY22*) and control (TRV:00) pepper leaves under fluorescent modulation meter; (**F**) phenotypic effect of *R. Solanacearum* treatment on *CaWRKY22-*silenced (TRV:*CaWRKY22*) and control (TRV:00) pepper plants at 7 dpi; (**G**) qRT-PCR analysis of transcriptional levels of defense-related marker genes in *CaWRKY22-*silenced (TRV:*CaWRKY22*) and control (TRV:00) pepper plants 24 h post inoculation with *R. Solanacearum*. The relative expression level of mock treated unsilenced plants was set to “1”. Error bars indicated the standard error. Data represents the means ± SD from four biological replicates. Different letters indicate significant differences, as determined by Fisher’s protected LSD test: uppercase letters, *p* < 0.01; lower case letters, *p* < 0.05.

The loss-of-function experiment was performed in pepper seedlings by Virus Induced Gene Silencing (VIGS) to investigate the role of *CaWRKY22* in immunity. 50 plants of TRV:00 and 50 plants of TRV:*CaWRKY22* were acquired. Six plants from the TRV:*CaWRKY22* plants were randomly selected to assess their gene silencing efficiency by root inoculation with cells of the virulent *R. Solanacearum* strain FJC100301. The result showed that in *R. Solanacearum* challenged TRV:*CaWRKY22* pepper plants, transcriptional levels of *CaWRKY22* were reduced to ~30% of that in TRV:00 plants, showing the successful silencing of *CaWRKY22* (Figure 4A). After *R. Solanacearum* inoculation, TRV:*CaWRKY22* pepper plants exhibited a significantly enhanced susceptibility to the pathogen compared to TRV:00 (control). This susceptibility was coupled with an increase in the growth of *R. Solanacearum* in *CaWRKY22*-silenced pepper plants, manifested by higher cfu values compared with those in the control plants at 3 dpi (days post inoculation) (Figure 4B). Histochemical staining was performed to assess cell death and H_2_O_2_ production in *R. Solanacearum*-infected, *CaWRKY22*-silenced, and control pepper leaves, an intensive DAB (dark brown color) staining (indicator of H_2_O_2_ accumulation) and hypersensitive reaction (HR) mimic cell death, manifested by darker trypan blue staining, were detected in the control leaves at 48 hpi, whereas the intensities of DAB and trypan blue staining were distinctly reduced in *CaWRKY22*-silenced leaves (Figure 4C). Ion leakage was estimated to analyze the severity of cell death and plasma membrane damage after inoculation by *R. Solanacearum*, the results showed that the unsilenced pepper plants exhibited higher ion leakage compared to *CaWRKY22-*silenced pepper plants at 24, 48, and 72 hpi (Figure 4D). A fluorescent modulation meter was used to take the photos of leaf cell death in TRV:*CaWRKY22* and TRV:00 after infection with *R. Solanacearum* FJC100301. The cell death detected in the unsilenced pepper leaves was very noticeable and strong, while very weak and negligible cell death was detected in *CaWRKY22*-silenced plants leaves (Figure 4E). 10 pepper plants of TRV:*CaWRKY22* and TRV:00 were randomly selected and root inoculated with *R. Solanacearum*. At 7 dpi, definite wilting symptoms were observed in *CaWRKY22*-silenced pepper plants, whereas unsilenced plants exhibited only faint wilting symptoms (Figure 4F). QRT-PCR was used to check the transcriptional expression levels of known defense-related genes, the results showed that transcriptional levels of the defense-related pepper genes, including *CaPO2*, *CaPR4*, *CaACC*, *CaBPR1*, *CaDEF1*, and *CaHIR1*, were lessened in *CaWRKY22*-silenced pepper plant leaves compared to that in control pepper plants at 24 hpi (Figure 4G).

### 2.5. Transient Overexpression of CaWRKY22-Triggered HR-Like Cell Death and Accumulation of H_2_O_2_ in the Leaves of Pepper Plants 

**Figure 5:**
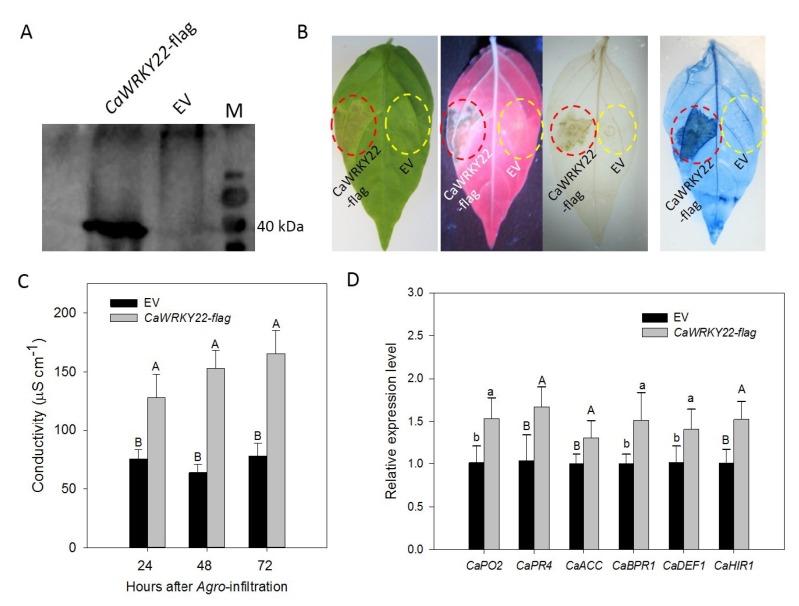
Transient over expression of *CaWRKY22* in pepper leaves triggered intense HR-like cell death and ROS accumulation. (**A**) Western blotting was performed to confirm the successful overexpression of CaWRKY22-Flag; (**B**) HR caused by transient overexpression of 35S:*CaWRKY22*, confirmed by phenotype detection, UV light exposure, and DAB and Trypan Blue staining at 4 dpi, respectively; (**C**) measurement of electrolyte leakage (ion conductivity) to evaluate the cell death response in leaf discs at 24, 48, and 72 h post agro-infiltration, respectively; (**D**) qRT-PCR analysis of the expression of immunity-related marker genes, including *CaPO2*, *CaPR4*, *CaACC*, *CaBPR1*, *CaDEF1*, and *CaHIR1*, in 35S:*CaWRKY22* expressed pepper leaves at 24 hpi, respectively. The relative expression level of marker genes in pepper leaves transiently expressing the empty vector were set to “1”. Data represent the means ± SD from four biological replicates. Error bars indicated the standard error. Different letters above the bars shows significant differences between the means, as analyzed by Fisher’s protected LSD test: uppercase letters, *p* < 0.01; lower case letters, *p* < 0.05.

The results of the *CaWRKY22* silencing experiment indicate that *CaWRKY22* acts as a positive regulator in pepper’s response to RSI. To further confirm this speculation, a transient overexpression assay was performed in pepper leaves to investigate the effect of *CaWRKY22* transient overexpression on the induction of plant HR cell death. A Western blotting assay confirmed that CaWRKY22 was successfully expressed in pepper plants (Figure 5A). An intensive cell death was manifested by darker trypan blue staining, and a higher level of accumulation of H_2_O_2_ displayed with darker DAB staining was detected in pepper leaves infiltrated with *Agrobacterium* cells carrying 35S:*CaWRKY22* compared to the control plant leaves infiltrated with *Agrobacterium* cells carrying 35S:00 (Figure 5B). Consistently, the pepper leaves transiently overexpressing *CaWRKY22* exhibited significantly higher ion leakage at 24, 48, and 72 h post inoculation compared to the leaves expressing the empty vector (Figure 5C). QRT-PCR was performed to examine the relative transcriptional levels of defense-related genes including the SA-responsive *CaPR4* and *CaBPR1*, JA-responsive *CaDEF1*, ET biosynthesis-associated *CaACC*, HR marker *CaHIR1*, and ROS detoxification-associated *CaPO2*. The results showed that *CaWRKY22* transient overexpression in pepper leaves significantly increased the transcriptional levels of *CaBPR1*, *CaPR4*, *CaDEF1*, *CaACC*, *CaHIR1*, and *CaPO2* as compared to that in pepper leaves transiently overexpressing empty vector (Figure 5D). All these data demonstrate that CaWRKY22 acts as a positive regulator of plant cell death.

### 2.6. CaWRKY22 Binds to the W-Box Containing Promoter Fragment but Not the Fragments without W-Box

**Figure 6:**
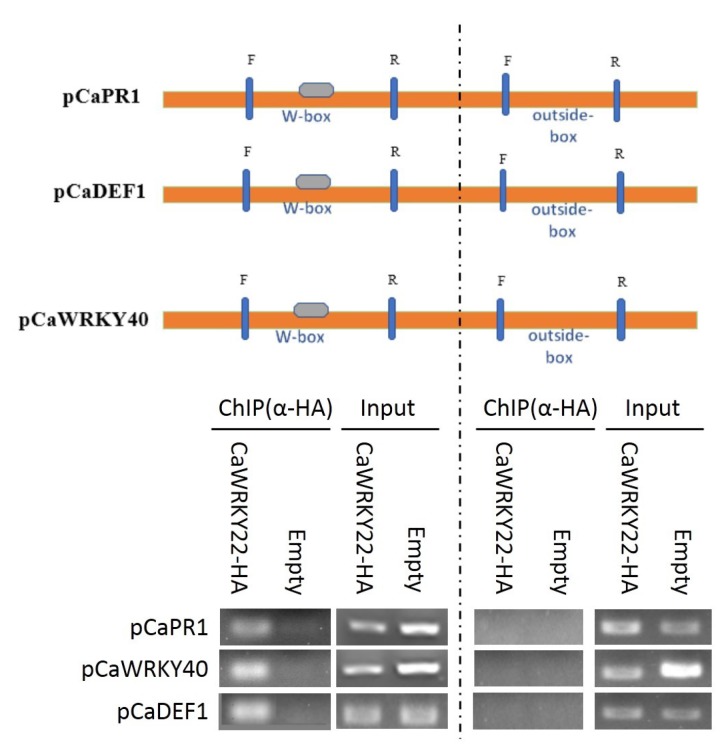
CaWRKY22 binds to the W-boxes of different marker genes as detected by chromatin immunoprecipitation (ChIP) analysis. CaWRKY22-HA was transiently overexpressed in pepper leaves, the chromatin was isolated, the DNA–protein complex was immunoprecipitated using anti-HA antibodies and adjusted to the same concentration. PCR was performed using primer pairs based on the sequence flanking the W-box within the promoters of *CaPR1*, *CaDEF1*, and *CaWRKY40*. “F” stands for forward while “R” represent reverse.

It has been generally reported that the majority of members of the WRKY family fulfill their functions by targeting and binding the conserved W-box [TTGAC(C/T)] present in the promoters of their target genes [23]. To test if CaWRKY22 can activate the transcription of its target genes in a W-box dependent manner, a chromatin immuno-precipitation (ChIP) assay was performed on chromatins isolated from *CaWRKY22*-HA which were transiently overexpressed in pepper leaves. The chromatin was sheared into fragments from 300 to 500 bps in length, and immuno-precipitated (IPed) with antibody of HA. The IPed DNA fragments were collected as a template for PCR with specific primer pairs of the conserved W-box containing promoter fragments of *CaPR1*, *CaDEF1*, and *CaWRKY40*. The results showed that CaWRKY22 could directly bind to the 250 bp W-box-containing fragments within the promoters of tested immunity associated genes, whereas no binding signal was detected in the promoter fragments within the promoter regions of the tested genes without W-box (Figure 6). These results indicate that CaWRKY22 binds to the W-box containing promoter regions, but does not bind to the promoter regions without W-box. In particular, CaWRKY40, a positive regulator in the pepper response to RSI, high temperature, and high humidity stresses, as identified in our previous study [9] , is also found to be a target of CaWRKY22.

### 2.7. The Inter-Relationship between CaWRKY22 and CaWRKY40

**Figure 7:**
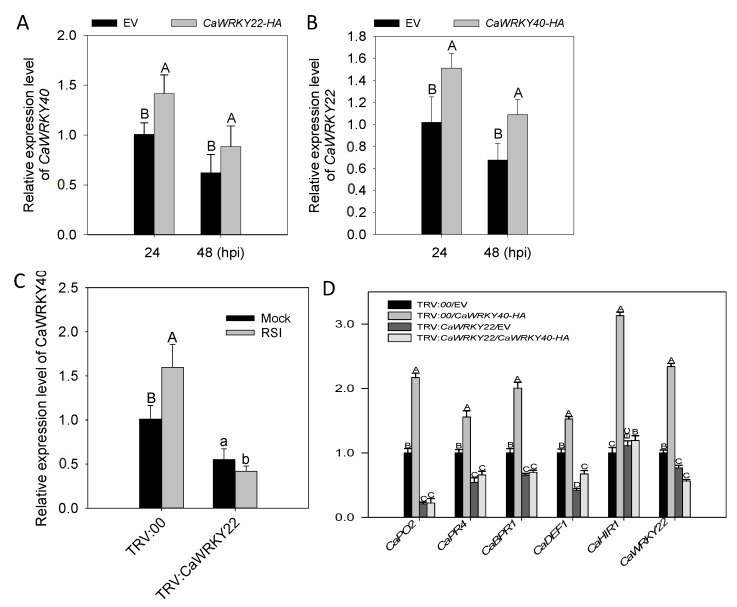
The inter-relationship between *CaWRKY22* and *CaWRKY40*. (**A**) Transcriptional expression of *CaWRKY40* in pepper leaves transiently overexpressing *CaWRKY22* at 24 and 48 hpi. The expression level of pepper leaves transiently overexpressing the empty vector was set to “1”; (**B**) transcriptional expression of *CaWRKY22* in pepper leaves transiently overexpressing *CaWRKY40* at 24 and 48 hpi. The expression level of pepper leaves transiently overexpressing the empty vector was set to “1”; (**C**) qRT-PCR analysis of *CaWRKY40* expression levels in *CaWRKY22*-silenced and control pepper plants. The expression level of mock treated unsilenced pepper leaves was set to “1”; (**D**) qRT-PCR analysis of the transcriptional levels of defense-related marker genes in *CaWRKY22*-silenced and control pepper plants transiently overexpressing 35S:*CaWRKY40-HA* and 35S:00. (**A**–**D**) Data represents the means ± SD from four biological replicates. The relative expression level of mock treated unsilenced plants was set to “1”. Error bars indicated the standard error. Different letters indicate significant differences between means, as determined by Fisher’s protected LSD test: uppercase letters, *p* < 0.01; lower case letters, *p* < 0.05.

As the promoter of *CaWRKY40* was bound by CaWRKY22, we speculated that *CaWRKY40* was transcriptionally regulated by *CaWRKY22*. To test this possibility, and to further assay the possible feedback regulation of *CaWRKY40* by *CaWRKY22*, QRT-PCR was employed to check the possible modulation of *CaWRKY22* by transient overexpression of *CaWRKY40*, or its silencing, as well as the possible effect of *CaWRKY22* transient overexpression or silencing on the expression of *CaWRKY40*. The results showed that transcription of *CaWRKY22* in *CaWRKY40*-transiently-expressing pepper leaves increased at 24 and 48 hpi compared to the control (Figure 7A). On the other hand, the transcriptional abundance of *CaWRKY40* was also increased in *CaWRKY22-*overexpressing leaves at 24 and 48 hpi (Figure 7B). By contrast, it was found that transcriptional levels of *CaWRKY40* were significantly downregulated in *R. Solanacearum* inoculated *CaWRKY22*-silenced pepper plants compared to that in the control plants (Figure 7C). In addition, *CaWRKY22*-silencing fully or partially suppressed the upregulation of the tested defense associated marker genes including *CaBPR1*, *CaPO2*, *CaPR4*, *CaDEF1*, and *CaHIR1*; triggered by *CaWRKY40* transient overexpression (Figure 7D). This suggests direct transcriptional regulation of *CaWRKY40* by *CaWRKY22* and that there exists a positive regulatory loop between *CaWRKY22* and *CaWRKY40*.

### 2.8 The Inter-Relationship between CaWRKY22 and CaWRKY6, CaWRKY27 and CaWRKY58

**Figure 8:**
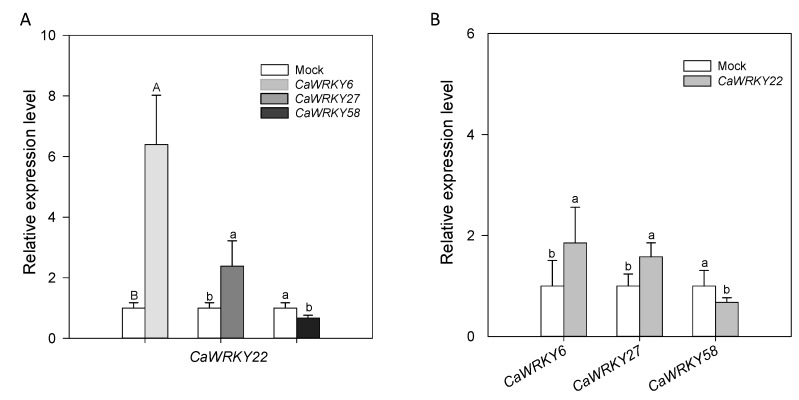
The inter-relationship between *CaWRKY22* and *CaWRKY6*, *CaWRKY27* and *CaWRKY58*. (**A**) Transcriptional expression of *CaWRKY22* in pepper leaves transiently overexpressing *CaWRKY6*, *CaWRKY27*, and *CaWRKY58* at 24 hpi. The expression level of *CaWRKY22* in pepper leaves transiently overexpressing the mock (empty vector) was set to “1”; (**B**) transcriptional expression of *CaWRKY6*, *CaWRKY27*, and *CaWRKY58* in pepper leaves transiently overexpressing *CaWRKY22* at 24 hpi. The relative expression level of target genes in mock treated plants (empty vector) was set to “1”. Data represents the means ± SD from four biological replicates. Error bars indicated the standard error. Different letters on the bars indicate significant differences between means, as determined by Fisher’s protected LSD test: uppercase letters, *p* < 0.01; lower case letters, *p* < 0.05.

As *CaWRKY40* was previously found to be expressionally and functionally related to other WRKYs, including *CaWRKY6*, the close relationship between *CaWRKY22* and *CaWRKY40* implies that *CaWRKY22* might also be associated with other WRKY TFs in pepper immunity against RSI. To test this speculation, the relationship between *CaWRKY22* and *CaWRKY6*, *CaWRKY27* and *CaWRKY58*, which have been implicated in pepper immunity against RSI by our previous studies, were assayed by qRT-PCR, the results showed that the transcriptional levels of *CaWRKY22* were increased in *CaWRKY6* and *CaWRKY27* transiently overexpressing leaves but were decreased in *CaWRKY58*-expressing leaves at 24 hpi (Figure 8A). On the other hand, the transcriptional levels of *CaWRKY6* and *CaWRKY27* increased while that of *CaWRKY58* decreased in *CaWRKY22*-expressing leaves at 24 hpi (Figure 8B). These results suggest that *CaWRKY22* and *CaWRKY6*, *CaWRKY27*, and *CaWRKY58* are expressionally and functionally interrelated.

## 3. Discussion

WRKY proteins constitute one of the largest TF families in plants. A subset of members in this family, which have been found in *Arabidopsis* and rice, participate and play important roles in the regulation of plant immunity. Significant functional divergence among close structural homologs of WRKY proteins from different plant species has been already described [12], and the roles of WRKY TFs in plant immunity in non-model plants such as pepper remain to be elucidated. Our present study functionally characterized CaWRKY22, an IIe WRKY TF in pepper, and our data indicates that CaWRKY22 is a positive regulator in the response of pepper to RSI and a component of a WRKY transcriptional network including *CaWKRY6*, *CaWRKY27*, *CaWRKY40*, and *CaWRKY58*.

The involvement of *CaWRKY22* in pepper immunity is implied by the presence of pathogen responsive *cis*-elements such as TCA, TGACG-motif, and W-box in the promoter of *CaWRKY22*, and was further supported by the data that *CaWRKY22* transcriptional levels were significantly upregulated after RSI. Since the genes upregulated by a given stress have been frequently found to play roles in response to that stress [24], we postulated that *CaWRKY22* might act as a positive regulator in pepper resistance to RSI. This hypothesis was confirmed by data from loss-of-function of CaWRKY22 by VIGS and gain-function analyses by transient overexpression, respectively. The silencing of *CaWRKY22* by VIGS significantly increased the susceptibility of pepper plants to RSI. This impaired immunity was consistent with the enhanced growth of the inoculated *R. Solanacearum* and downregulated HR-associated *CaHIR1* [25,26,27], ROS scavenging related *CaPO2* [28], SA-dependent *CaBPR1* [29], *CaPR4* [30,31], JA-associated *CaDEF1* [32,33,34,35], and ethylene-dependent *CaACC* [36]. In contrast, the transient overexpression of *CaWRKY22* significantly triggered HR-like cell death and H_2_O_2_ accumulation, accompanied with upregulation of *CaHIR1*, *CaPO2*, *CaBPR1*, *CaPR4*, *CaDEF1*, and *CaACC*. These results strongly suggest a role of CaWRKY22 as a positive regulator in pepper cell death and immunity. Similarly, it has been previously found that *AtWRKY22*, a homologue of CaWRKY22 in Arabidopsis, functions as a positive regulator in pattern-triggered immunity (PTI) triggered by flg22, chitin, or submergence modulating by the MAP kinase cascade (MEKK1, MKK4/MKK5, and MPK3/MPK6) [37,38,39,40]. It can be inferred that, upon attack by *R. Solanacearum*, *CaWRKY22* is upregulated and therefore decreases the susceptibility of pepper plants to RSI.

Hormones such as SA, JA, and ET are involved in plant immune signaling networks. SA activates resistance against biotrophic pathogens, while JA and ET are generally important for immunity to necrotrophic pathogens [41]. Frequently the production of SA, JA, and ET is coupled with ETI or PTI. Depending on their concentrations, these phytohormones can act either synergistically or antagonistically during defense signaling [42,43]. Synergistic relationships among the three signaling components have been found in PTI. Compensatory relationships among the sectors have been found in ETI [44,45]. *CaWRKY22* was consistently found to be induced by exogenous application of SA, MeJA, or ETH. The tested SA-, JA-, and ET-dependent immunity associated marker genes, SA-dependent *CaBPR1* [29] and *CaPR4* [30,31], JA-associated *CaDEF1* [32,33,34,35] and ethylene-dependent *CaACC* [37], were all downregulated by silencing of *CaWRKY22*, but upregulated by the transient overexpression of *CaWRKY22* in pepper plants, indicating that *CaWRKY22* participates in defense signaling which is synergistically mediated by SA, JA, and ET and, therefore, leads to PTI.

Genome-wide analyses indicated the participation of multiple WRKY TFs in plant immunity [46,47,48,49,50]. By functional genomic studies, *WRKY11*, *-17* [49], *-18* [50], *-25* [51], *-28* [52], *-33* [53], *-38* [54], *-45* [55], *-46* [56], *-53* [56], *-62* [54], *-70* [56], and *-75* [52] have been functionally characterized in *Arabidopsis* immunity, acting as either positive or negative regulators. These TFs have been suggested to integrate into a transcriptional network composed of positive and negative feedback loops and feed-forward modules [57,58,59]. However, the composition of these networks in different plant species remains poorly understood. Our previous studies found that CaWKRY6, CaWRKY27, and CaWRKY40 act as positive regulators in pepper’s response towards RSI [9,18,19], and that CaWRKY58 acts as a negative regulator in the immune process [20]. Our present study suggests that CaWRKY22 acts as a positive regulator in plant cell death and the response of pepper to RSI, its transcription was upregulated by transient overexpression of *CaWRKY6 -27* and *-40*, while down regulated by the transient overexpression of *CaWRKY58*. On the other hand, the transient overexpression of *CaWRKY22* also upregulated the expressions of *CaWKRY6*, *CaWRKY27*, and *CaWRKY40*, while downregulated the expression of *CaWRKY58*, suggesting the existence of WRKY networks and positive feedback loops between *CaWRKY22* and *CaWRKY6*, *CaWRKY27* or *CaWRKY40*. Similar positive feedback loops are believed to be present in plant immunity [60]. Alike positive feedback loops have been found between CaWRKY40 and CaCDPK15, CaWRKY40 and CabZIP63, CaWRKY40 and CaWRKY6. By chromatin immuno-precipitation, CaWRKY22 was found to bind the promoter of *CaWRKY40*. Similarly, CaWRKY6 [9] and CabZIP63 [21] have also been found to directly and transcriptionally regulate the expression of *CaWRKY40* during pepper response to RSI. In light of these evidences, it can be inferred that CaWRKY40 might be orchestrated by multiple TFs. However, unlike *CaWRKY6* and *CabZIP63*, which were upregulated by exogenous application of ABA and phenocopy *CaWRKY40* in response to RSI or HTHH, *CaWRKY22* was downregulated by exogenously applied ABA and its silencing or transient overexpression in pepper plants did not exhibit any phenotypic effect on thermotolerance or the expression of thermotolerance-associated marker genes in the present study. ABA is implicated in the plant response to heat stress and drought stress. As a crucial signaling molecule [61,62,63,64], ABA affects plant immunity antagonistically [65,66,67,68]. In conclusion, the expression of *CaWRKY40* is coregulated at the transcriptional level by CaWRKY6, CabZIP63, and CaWRKY22 upon the challenge of RSI, and is regulated by CaWRKY6 and CabZIP63 but not CaWRKY22 when pepper plants are exposed to heat stress.

**Figure 9:**
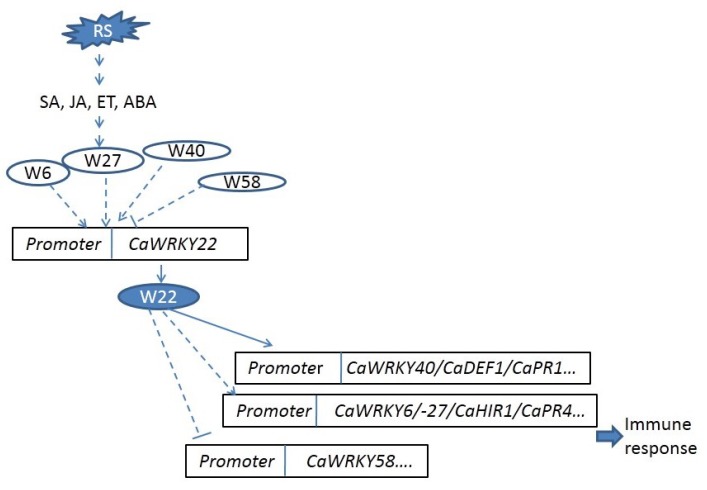
The proposed model of CaWRKY22 functions in pepper immunity against *Ralstonia Solanacearum*. RS: *Ralstonia Solanacearum*; W6: CaWRKY6; W22: CaWRKY22; W27: CaWRKY27; W40: CaWRKY40; W58: CaWRKY58. The dashed lines: indirect or direct regulation; the full line: direct regulation. 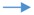. Positive regulation, 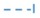. Negative regulation

A functional model of CaWRKY22 in the pepper immune response to *R. Solanacearum* was proposed, based on the data in the present study (Figure 9). When pepper plants are challenged by *Ralstonia Solanacearum*, SA, JA, and ET mediated signaling is activated, while ABA signaling is depressed. These signaling pathways might be transmitted to the nuclei where they are integrated in some way by various TFs including *CaWRKY6*, *-27*, *-40*, *or -58*. These proteins modulate directly or indirectly the transcription of *CaWRKY22*. CaWRKY22 in turn activates the transcription of *CaWRKY6*, *CaWRKY27*, and *CaWRKY40*, while depressing the transcription of *CaWRKY58*, therefore, CaWRKY22 activates pepper immunity against *R. Solanacearum*, forming WRKY networks with CaWRKY6, CaWRKY27, CaWRKY40, and CaWRKY58.

## 4. Experimental Procedures

### 4.1. Plant Materials and Growth Conditions

The seeds of Pepper (*Capsicum annuum*) cultivar GZ03 and *Nicotiana benthamiana* were procured from the pepper breeding group at the Fujian Agriculture and Forestry University (www.fafu.edu.cn). The seeds were sown in a soil mix (peat moss: perlite; 2/1, *v*/*v*) in plastic pots and placed in a greenhouse, and grew in a growth room at 25 °C, 60–70 µmol photons m^−2^·s^−1^, a relative humidity of 70%, and under a 16-h light/8-h dark photoperiod.

### 4.2. Vectors Construction

The vectors were constructed using the gateway technique. The full length ORF of *CaWRKY22* (with or without termination codon) was cloned into the entry vector pDONR207 by BP reaction to generate satellite vectors, and then transferred into destination vectors pMDC83, CD3688 (Flag-tag), pK7WG2, and CD3687 (HA-tag) to construct vectors for overexpression, subcellular localization, and ChIP assay by LR reaction, respectively. To prepare vectors for VIGS, a 308 bps fragment in the 3′-untranslated region (UTR) of *CaWRKY22* was selected, and the specificity was confirmed by a BLAST against the genome sequence in database of CM334 (http://peppergenome.snu.ac.kr/) and Zunla-1 (http://peppersequence.genomics.cn/page/species/blast.jsp). The specific fragment was cloned into the entry vector pDONR207 by Briggs–Rauscher (BR) reaction and then into the PYL279 vector by LR reaction.

### 4.3. Pathogens and Inoculation Procedures

*R. Solanacearum* strain FJC100301 was isolated from disease affected pepper plants from Fujian province (China) [19]. By using the tetrazolium chloride method stem exudates were purified [68]. *R. Solanacearum* was cultured in SPA medium (200 g potato, 20 g sucrose, 3 g beef extract, 5 g tryptone, and 1 L of double-distilled H_2_O) overnight at 28 °C and 200 rpm. The cultured *R. Solanacearum* was centrifuged and the pellet was suspended in distilled sterilized 10 mM MgCl_2_. Bacterial cell density was adjusted to 10^8^ cfu·mL^−1^ (OD600 nm = 0.8). To assay the effect of *R. Solanacearum* inoculation on transcription of *CaWRKY22* and the resistance of pepper to RSI, pepper plants were inoculated with 10 µL *R. Solanacearum* into the top third leaf with the help of a syringe without a needle. The respective leaves were harvested at the indicated time points for RNA extraction and histochemical staining, including DAB and trypan blue staining. To study the *CaWRKY22-*silenced phenotype in pepper plants under *R. Solanacearum* inoculation, the roots were injured by giving little cuts with a glass rod and then inoculated with the *R. Solanacearum*. After inoculation, the plants were grown in a growth chamber at 28 ± 2 °C, 60–70 µmol photons m^−2^·s^−1^, relative humidity of 70%, and under a 16-h light/8-h dark photoperiod.

### 4.4. Plant Treatments with Exogenously Applied Phytohormones 

For the phytohormone treatments, the healthy pepper plants at the four-leaf stage were sprayed with 1 mM SA, 100 µM methyl jasmonate (MeJA), 100 µM ABA, and 100 µM ETH, respectively. Mock plants were sprayed with sterile ddH_2_O. The treated samples above were harvested for RNA extraction at the indicated time points.

### 4.5. Analysis of CaWRKY22 Subcellular Localization

*Agrobacterium* containing *35S:CaWRKY22-GFP* or *35S:GFP* (used as control) were cultured overnight in a LB medium containing the corresponding antibiotics. Bacteria were centrifuged and the pellet was suspended in induction medium (10 mM 2-Morpholinoethanesulfonic acid [MES], 10 mM MgCl_2_ pH = 5.4, and 200 μM acetosyringone), and adjusted to OD_600_ = 0.8. *Agrobacterium* cells harboring the *35S:CaWRKY22-GFP* and *35S:GFP* were infiltrated into *N. benthamiana* leaves with a needleless syringe. 4′,6-Diamidino-2-phenylindole (DAPI) staining was performed as described previously [20] to specifically stain the nuclei. GFP and DAPI fluorescence signals were detected and images were taken using a Leica fluorescence light microscope (Tokyo, Japan) with an excitation wavelength of 488 nm, a band-pass emission filter, and an excitation wavelength of 405 nm with a 435–480 nm band-pass emission filter.

### 4.6. Histochemical Staining

Trypan blue and 3,3′-diaminobenzidine (DAB) staining was performed according to the previously published method of [21,22,69]. For trypan blue staining, pepper leaves were boiled in trypan blue solution (10 mL lactic acid, 10 mL glycerol, 10 mL phenol, 40 mL ethanol, 10 mL ddH_2_O, and 1 mL trypan blue) for 20 min, kept at room temperature for 8 hours, then placed into a chloral hydrate solution (2.5 g of chloral hydrated dissolved in 1 mL of distilled water) and boiled for 25 min for destaining; this process was repeated in triplicate. Finally, samples were kept in 70% glycerol. For DAB staining, the leaves were immersed in 1 mg/mL of DAB solution and maintained at room temperature overnight. Lactic acid:glycerol:absolute ethanol [1:1:3 (v/v/v)] solution was used to destain the DAB-stained pepper leaves and kept in 95% absolute ethanol [70]. A camera and light microscope (Leica, Wetzlar, Germany) were used to take the images of trypan blue and DAB staining.

### 4.7. Virus-Induced Gene Silencing (VIGS) of CaWRKY22 in Pepper Plants

The Tobacco Rattle Virus (TRV)-based virus induced gene silencing (VIGS) system was employed for *CaWRKY22* silencing in pepper plants, following the method of our previous studies [21,22,71,72]. *Agrobacterium* cells containing TRV1 and TRV2, TRV2-*CaWRKY22*, or TRV2-*PDS* (OD_600_ = 0.8) constructs were mixed in a 1:1 ratio, respectively. This mixture was infiltrated into cotyledons of 2-week-old pepper plants using a syringe without a needle. The *Agrobacterium*-inoculated pepper plants were grown in a growth chamber at 16 °C in the dark for 56 h with 45% relative humidity, and then transferred into a growth room at 25 ± 2 °C, 60–70 µmol photons m^−2^·s^−1^ and a relative humidity of 70%, under a 16-h light/8-h dark cycle.

### 4.8. Transient CaWRKY22 Expression Assay

*Agrobacterium* cells containing the 35S:*CaWRKY22-flag* vector were cultured to OD600 = 1.0 in LB medium containing the corresponding antibiotics overnight. These cells were then centrifuged at 7800 rpm, 28 °C for 10 min. The pellet was suspended into the induction medium (10 mM MES, 10 mM MgCl_2_, pH 5.4, and 200 μM acetosyringone) and adjusted to OD_600_ = 0.8. The suspension was infiltrated into pepper leaves using a needleless syringe. Later, the infiltrated plants were regularly observed for HR cell death, and harvested for trypan blue or DAB staining. RNA extraction used for detection of the expression of immunity associated genes.

### 4.9. Total RNA Isolation and qRT-PCR 

Total RNA was extracted from pepper leaves samples and wild type seedlings, using TRIzol reagent (Invitrogen, Carlsbad, CA, USA), were reverse transcribed by using the PrimeScript RT-PCR kit (TaKaRa, Dalian, China). To determine the relative transcription level of targeted genes, qRT-PCR and the corresponding data processing were performed, according to the method used in our previous studies [17,21,22,72], with specific primers (see Table S2) using the Bio-Rad Real-time PCR system (Bio-Rad, Foster City, CA, USA) and SYBR premix Ex Taq II system (TaKaRa Perfect Real Time).

### 4.10. Measurement of Ion Conductivity

Ion leakage was measured as previously described with slight modifications [9]. The six leaf discs (4 mm in diameter) were cut with a hole-punch, washed with sterilized ddH_2_O thrice, and immediately incubated in 20 mL of double distilled water. Discs were kept in a shaker with gentle shaking (60 rpm) for 1 h at room temperature. Ion conductivity was recorded using a conductivity meter (Mettler Toledo 326 Mettler, Zurich, Switzerland).

### 4.11. Chromatin Immuno-Precipitation Analysis (ChIP)

The ChIP assay was carried out according to a previously described protocol [9]. The *Agrobacterium* cells carrying 35S*:CaWRKY22*-HA or 35S*:CaWRKY40*-HA were infiltrated into the pepper leaves at the eight leaf stage. The infiltrated leaves were harvested at the required time point and ~2 g leaves of pepper were fixed with 1.0% formaldehyde for 5 min. The chromatin was sheared by sonication to an average length of 300–500 bp and immuno-precipitated with antibody against hemagglutinin (anti-HA; Santa Cruz Biotechnology, Dallas, TX, USA). The immuno-precipitated DNA was analyzed for enrichment of CaWRKY22 at the promoter region of targeted genes by common ChIP-PCR. The primers used for ChIP-PCR analysis are listed in Table S3.

## 5. Conclusions

The data in the present study indicates that CaWRKY22 targets to nuclei, is up-regulated by RSI and confer enhanced immunity against *Ralstonia solanccearum* by directly targeting *CaWRKY40* and incorporating into a WRKY network including *CaWRKY6*, *CaWRKY27*, *CaWRKY40*, and *CaWRKY58*.

## Supplementary Materials

Supplementary materials can be found at http://www.mdpi.com/1422-0067/19/5/1426/s1.
