# Supplementary material for: CaWRKY22 Acts as a Positive Regulator in Pepper Response to Ralstonia Solanacearum by Constituting Networks with CaWRKY6, CaWRKY27, CaWRKY40, and CaWRKY58
Source: Int J Mol Sci. 2018 May 10;19(5):1426. doi: 10.3390/ijms19051426 (PMC5983767; doi:10.3390/ijms19051426)
Supplement: Supplementary file 1 [file ijms-19-01426-s001.pdf]

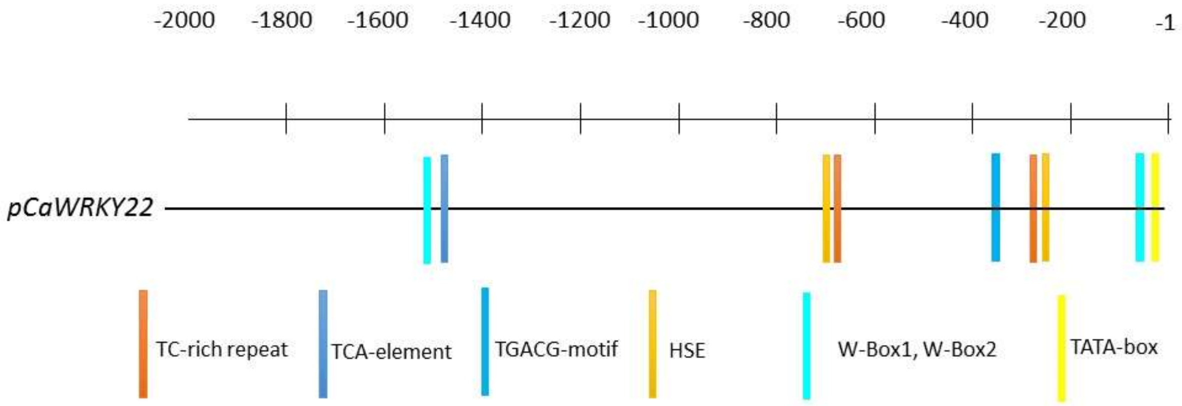

Supplementary Figure 1. Promoter *cis*-elements analysis of *CaWRKY22*

TCA-element: SA-relative element; TGACG-motif: MeJA-relative element; HSE: heat shock element; W-Box1, W-Box2: transcription factor WRKY binding element.

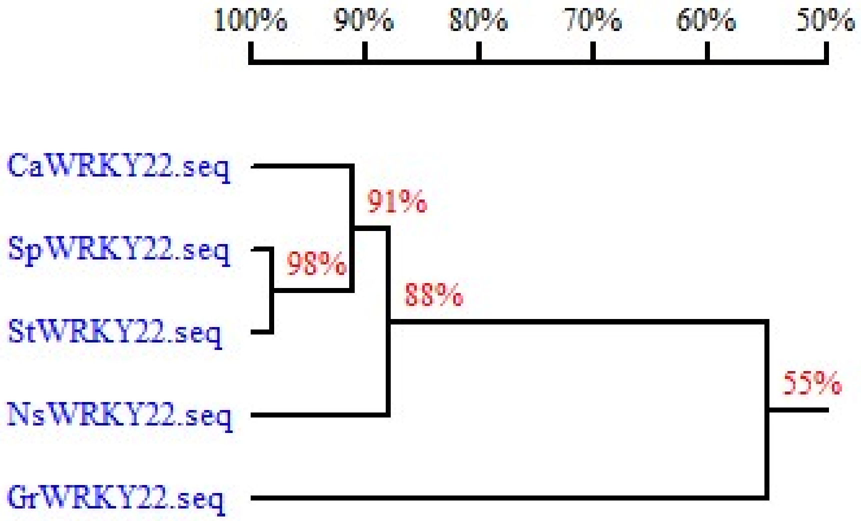

Supplementary Figure 2. Dendrogram presenting amino acid similarity of CaWRKY22 and WRKYs from other species

CaWRKY22 shares 91%, 91%, 88% and 55% amino acid identities to SpWRKY22, StWRKY22, NsWRKY22 and GrWRKY22 respectively.

Supplementary Table 1

Pepper primers used for vectors construction in this study

Gene	Forward primer (5'-3')	Reverse primer (3'-5')	Bps
CaWRKY22[1]	GGGGACAAGTTTGTACAAAAAAGCAGGC TTC ATGGAGGAAGATTGGGATCT	GGGGACCACTTTGTACAAGAAAGCTGGGT CTCAAACACCGCCAGCTGCGG	1122
CaWRKY22[2]	GGGACAAGTTTGTACAAAAAAGCAGGC GAGGCTGCACAGCTAGTTCCAC	GACCACTTTGTACAAGAAAGCTGGGTCGT CCACCAAGAACAGAGAGGGG	308
WRKY22[3]	GGGGACAAGTTTGTACAAAAAAGCAGGC TTC ATGGAGGAAGATTGGGATCT	GACCACTTTGTACAAGAAAGCTGGGTCAAC CCGCCAGCTGCGGTGG	1119

[1]**Primers for full length cloning of *CaWRKY22***

[2]**Primers for construction of TRV::*CaWRKY22* vector**

[3]**Primers for construction of 35S::*CaWRKY22-GFP***

Supplementary Table 2

Primers used in Real time RT PCR analyses for this study

Gene	Accession number	Forward primers (5'-3')	Reverse primers(3'-5')
CaWRKY22	CA08g07730	GAGGCTGCACAGCTAGTTCCAC	CACCAAGAACAGAGAGGGG
CaWRKY40	AAX20040.1	AACTTGGATGTTGTGCCTGGA	CTGTAACCTTGGCTTTTATGTGC
CaHIR1	AY529867	GACAAAGCTAATGAAGCATTCT AC	GGTGTCGAAGTACTGGGTTACC
CaPR4	AF244122.1	CAACCCGCAGAACATCAACTG G	CCTCAAGCATCTACCGCAAGCA
CaActin	GQ339766	AGGGATGGGTCAAAAGGATGC	GAGACAACACCGCCTGAATAGC
18srRNA	EF564281	CCGGTCCGCCTATGGTGTGCAC CGGTCGTC	GCAGTTGTTCGTCTTTCATAAAT CCAAGAA
CaDEF1	AF442388	GTGAGGAAGAAGTTTGAAAGA AAGTAC	TGCACAGCACTATCATTGCATAC AATTC
CaACCoxida se	AB434925.1	CCATTGTGGTCAACCTTGGC	GCATCGCTTCCTGGATTGTAA
CaPO2	DQ489711	GCCTATGCATTAATGTTGCAAA G	AGGCAGCATAACGAAGAATT
CaBPR1	AF053343	CAGGATGCAACACTCTGGTGG	ATCAAAGGCCGGTTGGTC

[1]Specific primers to detect relative expression of *CaWRKY22* designed according to the sequence in 3'UTR Specific primers for marker genes

Supplementary Table3

Pepper Primers Used for ChIP-PCR in This Study

**Gene**	**Forward primers (5'-3')**	**Reverse primers(3'-5')**
CaWRKY22 1W -p	TCATTTCTCGTCAAATAGAGTA	GCTGAATGGTGAGATAGTGGTA
CaWRKY22 2W -p	GAAACCAGCCAAATCACAAACA	GTTGACCTTC TTAGGGGATA GA
CaWRKY40 8/9W-p	CAAAAGTAGTTTCTGTTTCCAT	AGAGGCTCATCTGGGTGAATT
pCaPR1 1W-box	AGCTCCATCCCAAACCAACC	TGGTGTTGGGTCTGTGAGGC
pCaDEF1 1W-box	AATCAGTGCCGACTGTGGGG	GCGCACCTCGGCGCTGAGCT
CaWRKY-22 OUTSIDE	AAATGGAACGGAGGGAGTAGTA	TTGGACCTTTTCTTTTATGGGT
